# Examining BRITEpath, a Digital Intervention for Reducing Adolescent Suicide Risk in Primary Care: A Randomized‐Controlled Trial

**DOI:** 10.1111/sltb.70114

**Published:** 2026-06-09

**Authors:** Nermin Toukhy, Candice Biernesser, Jamie Zelazny, Brandie George‐Milford, Casey Monteverde, Giovanna Porta, Kaleab Z. Abebe, David Brent, Stephanie Stepp

**Affiliations:** ^1^ Department of Psychiatry, School of Medicine University of Pittsburgh Pittsburgh Pennsylvania USA; ^2^ Western Psychiatric Hospital University of Pittsburgh Medical Center Pittsburgh Pennsylvania USA; ^3^ Department of Medicine, School of Medicine University of Pittsburgh Pittsburgh Pennsylvania USA

**Keywords:** adolescents, digital health intervention, primary care, smartphone app, suicide risk

## Abstract

**Introduction:**

Previous research has identified a high need for suicide risk management in primary care settings among adolescents. This randomized controlled trial examined the efficacy and feasibility of BRITEpath, a smartphone‐based application and clinician portal designed to help youth in reducing suicide risk.

**Methods:**

Participants (*n* = 101, ages 12–26, 85.1% female) that were either referred to or were receiving mental health services in primary care were randomly assigned (2:1 allocation) to BRITEpath + TAU (*n* = 68) or TAU alone (*n* = 33). Independent evaluators, blinded to treatment, assessed outcomes at baseline, Week 4, and Week 12. Primary outcomes included suicidal thoughts and behaviors, depression, mental health service use, and quality of life; secondary outcomes examined usability and satisfaction.

**Results:**

No significant differences were found between BRITEpath + TAU and TAU for primary outcomes overall. However, BRITEpath participants reported lower suicidal thoughts over time. History of inpatient and outpatient care moderated the effect of BRITEpath on depression. Participants reported high satisfaction and perceived usefulness of the app.

**Conclusions:**

BRITEpath shows promising effects on suicidal thoughts and depression, particularly among those with prior psychiatric care. Future research should explore strategies to enhance engagement, such as integration with ongoing care, which may strengthen the impact on clinical outcomes.

**Trial Registration:**

ClinicalTrials.gov identifier: NCT04672798

## 1 Introduction

Rates of depression and suicidality among adolescents and young adults have been increasing in the US, with suicide being the second leading cause of death for those aged 10–24 (Ormiston et al. 2024; Shorey et al. 2022; Curtin and Garnett 2023). Primary care settings have a substantial and growing role in the detection and early intervention of depression and suicidality in adolescents. Studies have shown an upward temporal trend in the frequency with which adolescents screen positive for depression and suicidal risk in primary care visits (Davis et al. 2024; Lebrun‐Harris et al. 2022; Mayne et al. 2021). Furthermore, a study examining suicide deaths indicated that most had a primary care visit at the year prior to death (Braciszewski et al. 2023; Ahmedani et al. 2014).

While screening for depression and suicide thoughts and behaviors in primary care has become a standard of care, there are key challenges that need to be addressed. First, although there are certain interventions for depression in primary care settings (Aalsma et al. 2020), to the best of our knowledge, interventions for suicidality are limited (Aalsma et al. 2022). Thus, screening for suicidality without appropriate intervention embedded in primary care settings may result in increased referral to the emergency department and hospitalizations, which can be highly adverse situations for adolescents (Cadorna et al. 2024). Second, adolescents may be reluctant to seek professional help due to their preference for self‐reliance, posing a major barrier to mental health treatment (Radez et al. 2021). Similarly, a study indicated that only 26% of adolescents and young adults referred to mental health services due to suicidal concerns followed through with the referral (Aalsma et al. 2022).

Although not designed and tested specifically in primary care, several digital health interventions for the management of depression and suicidality in adolescents and young adults have been developed and evaluated in randomized controlled trials (RCTs). One such intervention is Therapeutic Evaluative Conditioning (TEC), a game‐like application designed to condition individuals to decrease identification of self with suicide and self‐injury, which has been shown to reduce non‐suicidal self‐injury and suicide attempts during the study, but the effects were not sustained at follow‐up (Franklin et al. 2016). Another example is Lifebuoy, a Dialectical Behavioral Therapy (DBT)‐based application designed to promote emotion regulation and distress tolerance, which showed significant improvements in Suicidal Ideation (SI) but not behaviors (Torok et al. 2022). iBobbly, a culturally adapted application designed for Australian aboriginal youth to facilitate emotional regulation, action planning and values identification, has been found to reduce depression but not SI or Suicidal Behavior (SB) (Tighe et al. 2017). BlueIce, an application designed for at‐risk adolescents based on CBT and DBT principles, although well accepted by adolescents, did not have a significant effect on suicidal ideation and behaviors (Stallard et al. 2024). In summary, previous digital health interventions focusing on suicide have yielded mixed findings, warranting further research.

Building on previous research (Goldstein et al. 2024; Kennard et al. 2018), we developed a digital health intervention, BRITEPath, developed to reduce suicide risk among adolescents and young adults vulnerable to suicide in primary care settings. BRITEpath was designed for delivery by mental health clinicians embedded in primary care settings to enhance their capacity to effectively manage depressed and suicidal adolescents. While other digitalized interventions focused on adolescents from community or mental health clinical settings, BRITEpath has the promise of addressing limits in mental healthcare accessibility in primary care settings by providing an easily accessible and personalized intervention for adolescents.

BRITEpath is composed of three components: (1) Guide2BRITE, a platform on which clinicians are guided in setting up the BRITE app, which includes developing personalized safety plans with youth; (2) BRITE, a smartphone application for adolescents that provides real‐time intervention in moments of distress; and (3) BRITEBoard, a web‐based portal for clinicians to monitor app engagement and clinical outcomes. Given that BRITEpath was designed for integration within clinician‐delivered care, the three components form a cohesive intervention model. Guide2BRITE serves as the entry point, guiding clinicians and patients in collaboratively developing a personalized safety plan that incorporates BRITE as a tool for ongoing self‐management. BRITE is then used by patients between sessions to provide just‐in‐time support during moments of distress, reinforcing coping strategies and safety planning. BRITEBoard is intended for use by clinicians to provide ongoing monitoring by tracking app engagement in between appointments (e.g., distress ratings, coping skills accessed) and enhanced symptom monitoring during appointments using clinician‐pushed assessments of clinical indicators (e.g., depression, suicidal ideation), thereby providing clinicians and patients with real‐time information on patterns of distress and app use. This information can be reviewed jointly during sessions to inform and refine the safety plan, reducing reliance on retrospective recall of distressing experiences, which can be particularly challenging among adolescents and young adults (Stone et al. 2003).

In this study, we examine the feasibility and the preliminary efficacy of BRITEPath on reducing suicide outcomes among adolescents and young adults who receive mental health services in primary care settings due to mood or behavioral problems. BRITE was previously developed as part of a safety‐planning intervention intended to reduce post‐discharge suicide attempts among adolescents hospitalized for suicidal thoughts and behaviors (Kennard et al. 2018). Previous studies have shown that BRITE is acceptable and usable for adolescents in inpatient settings and showed promise in reducing recurrent SB and preventing re‐hospitalization over the subsequent 6 months (Goldstein et al. 2024; Kennard et al. 2018). Thus, this study aims to extend prior work conducted in psychiatric inpatient settings (Goldstein et al. 2024; Kennard et al. 2018) into pediatric primary care.

### 1.1 Current Study

The present study is a single‐blinded, longitudinal RCT designed to examine the preliminary efficacy and feasibility (perceived usability and satisfaction) of BRITEPath and Treatment As Usual (TAU) on suicide‐related and clinical outcomes (SIBs, depression, utilization of mental health services, and quality of life) compared to TAU alone among adolescents that were either referred to or were receiving mental health services in pediatric primary care. More specifically, we hypothesize that those assigned to BRITEPath compared to TAU will endorse less SIBs and depression, higher quality of life, and will have higher rates of utilization of mental health services during follow‐ups. We also hypothesize that participants will find BRITEPath easy to use and will report high satisfaction with the intervention.

## 2 Methods

### 2.1 Participants

Eligible adolescents and young adults with mood or behavioral difficulties were identified by mental health (MH) clinicians embedded in four primary care clinics. Interested individuals were contacted by study clinicians for consent. Informed assent (for participants under 18) and consent (for those 18 and older) were obtained from participants and parental permission was obtained from guardians for those under 18. The study was approved by the University of Pittsburgh Institutional Review Board.

Inclusion criteria were: ages 12–26, referral to or receipt of services from a primary care MH clinician, access to a smart device (e.g., smartphone, iPad, tablet), and fluency in English. Exclusion criteria included conditions that impaired participation in assessments or intervention, current manic or psychotic symptoms, intellectual or developmental disorder, or life‐threatening medical conditions requiring immediate treatment, including imminent suicide risk.

### 2.2 Study Design

This study was preregistered under clinical trials and follows the standard Consolidated Standards of Reporting Trials (CONSORT) guidelines (Moher et al. 2001) for reporting (See CONSORT checklist in Table S1).

#### 2.2.1 Randomization

Upon completing baseline assessment, participants were randomly assigned using a computer‐based mechanism to BRITEpath + TAU or TAU alone in a 2:1 allocation, based on Efron's biased coin toss (Dumville et al. 2006; Efron 1971). Interventions in the two groups were received in parallel. As described previously by Begg and Iglewicz (1980), we balanced participants by sociodemographic and clinical characteristics, both within and across sites.

#### 2.2.2 BRITEpath

BRITEpath comprises three components: (1) Guide2BRITE; (2) BRITE; and (3) BRITEBoard. Participants assigned to BRITEpath + TAU received access to all three.

*Guide2BRITE* is an interactive website used by study clinicians to orient participants to BRITE and co‐develop a digital safety plan tailored to the participant's emotional needs and risk profile. The clinician guided youth through identifying emotional triggers, selecting coping strategies, and listing crisis resources. Although originally intended for use by embedded mental health (MH) clinicians in primary care, due to limited availability, this component was administered by study clinicians only.

*BRITE* is a smartphone application designed to help adolescents prevent or manage crises related to depression and SIBs through safety planning, distress tolerance, and emotion regulation strategies. Once downloaded, the app provided personalization options (e.g., uploading music, photos, and sharing contacts). Participants were prompted to complete daily distress ratings and were provided with evidence‐based coping tools, including savoring positive experiences, reviewing reasons for living, engaging with social contacts for distraction or support, and reaching out to professional and personal contacts during crises (Goldstein et al. 2024; Kennard et al. 2018). The app's development is described in detail elsewhere (Kennard et al. 2018).

*BRITEBoard* is a web‐based portal used to monitor app engagement (e.g., distress ratings, coping skills accessed) and clinical outcomes (e.g., depression, suicidal ideation). Data could be shared with MH clinicians as part of ongoing care; however, no clinicians accessed BRITEBoard during the study.

#### 2.2.3 Treatment as Usual

Both treatment groups received standard mental health care within pediatric primary care practices by the embedded MH clinicians, usually occurring virtually. This included assessment and referral for mental health treatment, as well as standard paper‐based safety‐planning for adolescents and young adults vulnerable to suicide and depression.

### 2.3 Measures

Sociodemographic information was collected from each participant and their parents at baseline assessment. This included participants' age (in years), sex at birth (male/female), and their self‐reported ethnicity, race, gender identity, and family income. Ethnicity, race, family income, and Sexual and Gender Minority (SGM) were dummy coded as Hispanic, White, < $75,000 per year, and SGM status, respectively.

#### 2.3.1 Primary Outcome Measures

##### 2.3.1.1 Suicidal Thoughts and Behaviors (STBs)

Participants' STBs were assessed at baseline (lifetime and past 3 months) and follow‐ups (since last assessment), using the Columbia Suicide Severity Rating Scale (C‐SSRS), a semi‐structured interview (Posner et al. 2011). The most severe suicide ideation (SI) score was used to reflect the highest level endorsed of SI, yielding a 5‐point ordinal scale ranging from: (1) passive SI, (2) non‐specific active SI, (3) active SI with method, (4) active SI with intent and (5) active SI with specific plan and intent. SB examines the occurrence of (1) preparatory suicidal acts as well as (2) aborted, (3) interrupted, and (4) actual suicide attempts. Any type of SB was used as an outcome in this study.

Suicidal thoughts were assessed using item 9 (“Thoughts that you would be better off dead, or of hurting yourself”) on the Patient Health Questionnaire‐9 (see details below), which refers to the frequency of passive and active suicidal thoughts (Razykov et al. 2012). Responses were scored on a Likert scale ranging from 0 (“not at all”) to 3 (“almost every day”). The suicidal thoughts measure was used as a binary outcome in this study, with 0 indicating no suicidal thoughts and 1 indicating any frequency of suicidal thoughts over the past 2 weeks.

##### 2.3.1.2 Depression

Depression was assessed using the Patient Health Questionnaire‐9 (PHQ‐9) (Kroenke et al. 2001), a nine‐item measure evaluating the frequency of depressive symptoms over the past 2 weeks at baseline and follow‐ups. Items are rated on a 4‐point Likert scale from 0 (“not at all”) to 3 (“almost every day”). The total PHQ‐9 score served as the primary depression outcome. The PHQ‐9 demonstrates strong psychometric properties in both adults (Kroenke et al. 2001) and adolescents (Richardson et al. 2010), and showed high internal consistency in this study (Cronbach's *α* = 0.837).

##### 2.3.1.3 Mental Health Services Utilization

The Child and Adolescent Service Assessment (CASA) (Ascher et al. 1996) measured past 3‐month mental health service utilization at baseline and since the last assessment at follow‐ups across six domains: psychiatric medication, outpatient treatment, inpatient treatment, school services (guidance/counseling), emergency department visits, and legal services (e.g., detention or jail). The 35‐item yes/no questionnaire was independently completed by both participants and their parents. A service was considered utilized if endorsed by either informant.

##### 2.3.1.4 Quality of Life and Social/Emotional Functioning

Quality of life was assessed using the 23‐item Pediatric Quality of Life Inventory Version 4 (PedsQL) (Varni et al. 2001), which measures five domains: Physical, Emotional, Social, and School Functioning. Items are rated on a 5‐point scale (from “almost always” to “never”), with higher scores indicating better quality of life. Domain scores were calculated as the sum of item ratings. The PedsQL has demonstrated strong psychometric validity and showed high internal consistency in this study (Cronbach's *α* = 0.915). As it was developed and validated for individuals under 18, it was administered only to participants below that age (*N* = 31).

#### 2.3.2 Secondary Outcome Measures

Utilization, usability, and satisfaction with the BRITE application (app) were assessed during an exit interview following study completion, using both multiple choice and open‐ended questions for more comprehensive feedback.

##### 2.3.2.1 Application Utilization

Application utilization was assessed by recording the number of participants who utilized each BRITE activity and the number of times each activity was used per participant.

##### 2.3.2.2 Usability and Satisfaction With the Application

Usability was assessed through five multiple‐choice items developed for the study, rated on a 7‐point scale (1 = strongly disagree to 7 = strongly agree), evaluating: (1) “BRITE's ease of use”; (2) “clarity of information on coping resources provided by BRITE”; (3) “clarity of rating levels of distress”; (4) “clarity of onboarding process”; and (5) “satisfaction with interacting with BRITE.” An average score on each item was used to represent usability scores. Satisfaction was measured using four adapted items from the Client Satisfaction Questionnaire (CSQ) (Larsen et al. 1979): (1) “I needed to learn a lot about the app before I could use it” (1 = strongly disagree to 5 = strongly agree); (2) “Would you recommend BRITE for a person in need?” (1 = no, definitely not to 4 = yes, definitely); (3) “How satisfied are you with the amount of help you received by the app?” (1 = very dissatisfied to 4 = very satisfied); and (4) “Did the app help you to deal more effectively with your problems?” (1 = seemed to make things worse to 4 = yes, a great deal). Each item was followed by an open‐ended question asking participants to elaborate on their response. Those were qualitatively analyzed for perceived usability, feasibility, acceptability, and efficacy.

### 2.4 Procedure

A clinically trained study clinician conducted a comprehensive baseline assessment with eligible participants, collecting data on sociodemographic factors, past and recent SIBs, depression, quality of life, and mental health service utilization. Following baseline, participants were randomized to BRITEpath + TAU or TAU alone. Those in the BRITEpath + TAU group were onboarded by the study clinician, who attempted to simulate how an embedded MH clinician would use the app and communicate with the participants' primary care team. This process included: (1) completing the Guide2BRITE, which involved developing a digital safety plan, identifying potential barriers to app engagement, and collaboratively problem‐solving with the participant and parents; (2) assisting participants with downloading and orienting to the BRITE app; and (3) reviewing the safety plan and available resources with parents. TAU followed the American Academy of Pediatrics (2023) guidelines for managing adolescent suicide risk in primary care, including standardized screening, safety assessment, and a paper‐based safety plan.

Follow‐up assessments at weeks 4 and 12 were conducted for both treatment groups by clinically trained independent evaluators, blinded to treatment condition. These evaluators (mental health clinicians), supervised for consensus on evaluations, assessed primary and secondary outcomes via phone interviews lasting 30–60 min. Participants received up to $100 in compensation ($20 at baseline, $30 at Week 4, and $50 at Week 12).

### 2.5 Statistical Analysis

Univariate group comparisons for measures at baseline were conducted using R (R Core Team 2021). Independent samples *t*‐test and Chi‐squared test were used to examine differences in primary outcomes and sociodemographic characteristics at baseline between those who received BRITEPath + TAU versus TAU. A Bonferroni correction was used to adjust for multiple comparisons due to multiple primary outcomes.

Mixed‐effects longitudinal analyses were conducted using Stata version 18 (2018, Stata Corp LP, Stata Statistical Software, College Station, TX) to determine whether there were changes in primary outcomes over time between those who were assigned to BRITEPath + TAU versus TAU. Models included the main effects of treatment group, time, and their interaction. Moderation was also tested for history of SIBs, and history of inpatient and outpatient care, by examining their interaction with treatment group and time. Time was defined as days since baseline to better represent the study duration for each participant. Mixed effects analysis was not used to examine quality of life as an outcome due to insufficient data (see measures above). Thus, we used independent samples *t*‐test to test for differences in this outcome by treatment group. Power analysis was conducted prior to data collection centering around the precision of confidence interval (CI) width estimation for feasibility outcomes. This revealed that a sample size of 100 (assuming 80% retention) affords us a 95% CI width of 0.09–0.13.

BRITE application utilization was analyzed by calculating the number of participants who engaged with each activity, as well as the average number of times each activity was used per participant. Usability was analyzed by the mean ratings of scores on the multiple‐choice usability items. As a secondary analysis, we also conducted univariate group comparisons between those who used the app compared to those who did not on primary outcomes.

Perceived usability, feasibility, acceptability, and efficacy were qualitatively analyzed using rapid qualitative methods (Nevedal et al. 2021). Two co‐authors (N.T. and C.M.) coded participants' responses using a templated worksheet in excel (See Codebook for Rapid Content Analysis in Table S2). Codes were informed by domains from the literature on usability, feasibility, acceptability, and efficacy (Zhong et al. 2023), while themes emerged inductively from the data. Coding differences were reviewed to reach consensus. Domains and themes were determined by the primary author. Themes for perceived usability include “ease of use, ease of access, problems with use and usefulness of app features”; for perceived feasibility themes include “adherence issues”; for perceived acceptability “satisfaction with application and perceived appropriateness”; and for perceived efficacy “help in general, help in moments of stress and help with symptoms” (detailed definitions of each domain and theme are presented in Codebook for Rapid Content Analysis in Table S2).

## 3 Results

**FIGURE 1:**
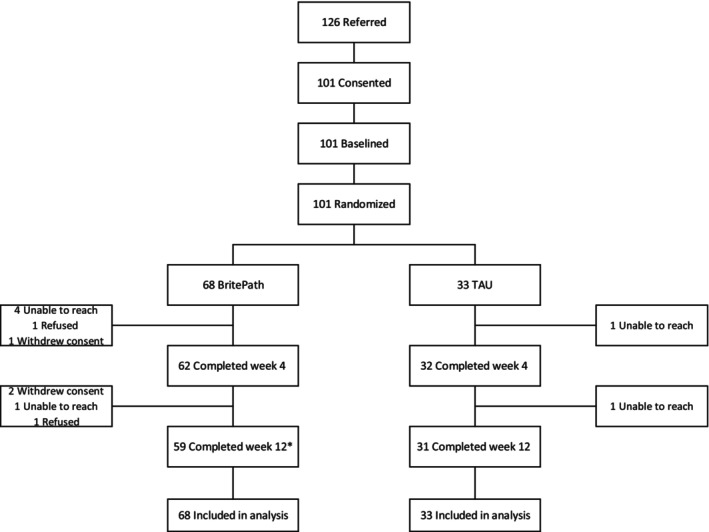
Consolidated Standards of Reporting Trials (CONSORT) diagram. *1 participant who did not complete week‐4 did complete week‐12.

Of the 126 participants referred to the study between 11/5/2020 and 07/31/2022, 101 provided consent to participate, completed baseline assessment, and were randomized. Sixty‐eight participants were assigned to BRITEPath and TAU and 33 to TAU alone (see Figure 1). The final sample had an average age of 19.24 (SD = 3.74), was mostly female at birth (85.1%), White (70.3%), and more than half identified as a sexual and/or gender minority (54.5%) (see Table 1).

**TABLE 1:** Sample sociodemographic and clinical characteristics by treatment group.

	*N*	Overall	BRITEPath	TAU	Test	Statistic
*N*		101	68	33		
Sociodemographic characteristics						
Age (range 12–26)	101	19.21 (3.74)	19 (3.77)	19.64 (3.70)	*t*(99)	0.801
Age (> 18)	101	59 (58.4%)	38 (55.9%)	21 (63.6%)	*𝜒* ^2^(1)	0.550
Sex (Male)	101	15 (14.9%)	11 (16.2%)	4 (12.1%)	*𝜒* ^2^(1)	0.029
Race (White)	101	71 (70.3%)	45 (66.2%)	26 (78.8%)	*𝜒* ^2^(1)	1.692
Ethnicity (Hispanic)	101	7 (7.0%)	4 (6.0%)	3 (9.1%)	*𝜒* ^2^(1)	0.331
Family income (> 75,000$)	59	25 (42.4%)	17 (45.9%)	8 (36.4%)	*𝜒* ^2^(1)	0.519
SGM	101	55 (54.5%)	40 (58.8%)	15 (45.5%)	*𝜒* ^2^(1)	1.601
Clinical characteristics						
Depression (PHQ total)	101	10.85 (5.85)	10.60 (5.95)	11.36 (5.71)	*t*(99)	0.611
Physical QOL (pedsq‐physical)	31	78.08 (19.78)	81.70 (19.00)	70.49 (18.01)	*t*(29)	−1.505
Emotional QOL (PedsQL‐emotional)	31	56.13 (24.69)	59.05 (27.09)	50.00 (18.41)	*t*(29)	−0.952
Social QOL (PedsQL‐social)	31	74.92 (24.97)	77.38 (23.75)	69.75 (27.95)	*t*(29)	−0.790
School QOL (PedsQL‐school)	31	59.52 (24.00)	63.33 (25.27)	51.50 (19.73)	*t*(29)	−1.300
Psychosocial QOL (PedsQL)	31	63.45 (20.67)	66.59 (22.88)	56.88 (13.77)	*t*(29)	−1.233
Overall QOL	31	68.54 (19.10)	71.84 (20.38)	61.60 (14.63)	*t*(29)	−1.419
Suicide thoughts and behavior^a^						
Suicidal thoughts (PHQ item 9)	101	0.48 (0.8)	0.50 (0.80)	0.42 (0.79)	*t*(99)	−0.447
Most severe ideation	101	1.23 (1.61)	1.37 (1.74)	0.94 (1.27)	*t*(99)	−1.398
Preparatory acts (CSSRS)	100	2 (2.0%)	1 (1.5%)	1 (3.0%)	*𝜒* ^2^(1)	0.267
Aborted attempt (CSSRS)	100	3 (3.0%)	3 (4.5%)	0 (0.0%)	*𝜒* ^2^(1)	1.523
Interrupted attempt (CSSRS)	100	2 (2.0%)	1 (1.5%)	1 (3.0%)	*𝜒* ^2^(1)	0.267
Actual attempt (CSSRS)	100	5 (5.0%)	5 (7.5%)	0 (0.0%)	*𝜒* ^2^(1)	2.592
Any suicial behavior	100	10 (10.0%)	8 (11.9%)	2 (6.1%)	*𝜒* ^2^(1)	0.849
Service utilization^b^						
Outpatient services	100	90 (90.0%)	61 (91.0%)	29 (87.9%)	*𝜒* ^2^(1)	0.246
School services	100	56 (56.0%)	36 (53.7%)	20 (60.6%)	*𝜒* ^2^(1)	0.424
Emergency services	100	14 (14.0%)	11 (16.4%)	3 (9.1%)	*𝜒* ^2^(1)	0.986
Legal services	100	1 (1.0%)	0 (0.0%)	1 (3.0%)	*𝜒* ^2^(1)	2.051
Inpatient services	100	25 (25.0%)	18 (26.9%)	7 (21.2%)	*𝜒* ^2^(1)	0.377
Medication services	98	83 (84.7%)	57 (86.4%)	26 (81.3%)	*𝜒* ^2^(1)	0.435

Abbreviations: QOL, quality of life; SGM, sexual gender minority.

^a^ Past‐3 months suicidal thoughts and behaviors assessed at baseline.

^b^ Past‐3 months service utilization assessed at baseline.

### 3.1 Sample Characteristics

No significant differences were found between groups in sociodemographic or baseline clinical variables (depression, quality of life), SIBs, or mental health service utilization (see Table 1). Of those who completed baseline assessments, 93% (*N* = 94) completed the 4‐week and 89% (*N* = 90) completed the 12‐week follow‐up. Participants with and without follow‐up data did not differ significantly in baseline sociodemographic characteristics, past mental health service use, quality of life, or SIB history (see Table S3). Those missing QOL data were more likely to have received past psychiatric medication treatment (75.3%, *N* = 64) compared to those with complete data (24.7%, *N* = 21; *χ* ^2^(1) = 12.20, *p* < 0.001). Mental health service utilization (medications, inpatient, outpatient or school services) did not differ significantly between groups at any timepoint (see Table S4). At baseline, the majority of the participants were receiving services (BRITEPath: 97.0%; TAU: 93.9%), with no significant group difference, *χ* ^2^(1) = 0.55, *p* = 0.461. Similarly, there were no significant differences at Week 4 (BRITEPath: 83.9%; TAU: 90.6%), *χ* ^2^(1) = 0.81, *p* = 0.369, or at Week 12 (BRITEPath: 86.4%; TAU: 81.3%), *χ* ^2^(1) = 0.43, *p* = 0.512.

### 3.2 Primary Outcomes

Table 2 shows results from mixed effects longitudinal analysis. There were no significant differences between the two groups in changes over time on most severe SI endorsed (*β* = −0.006, SE = 0.011, *p* = 0.549), presence of any SB (Odds Ratio (OR) = 0.022, SE = 0.029, *p* = 0.443), depression (*β* = −0.007, SE = 0.010, *p* = 0.483) and mental health services utilization outcomes [medication (OR = 1.004, SE = 0.011, *p* = 0.686), inpatient (OR = 0.989, SE = 0.023, *p* = 0.657), outpatient (OR = 0.999, SE = 0.010, *p* = 0.983) and school services (OR = 1.010, SE = 0.011, *p* = 0.397)]. None of the patients reported admission to the Emergency Room during follow‐up period. A significant main effect of time was observed for medication treatment utilization (OR = 0.969, SE = 0.010 *p* < 0.001), school (OR = 0.961, SE = 0.010, *p* < 0.001) and inpatient (OR = 0.963, SE = 0.019, *p* = 0.041) services, indicating a decrease in utilization of those services during the study in both groups.

**TABLE 2:** Longitudinal mixed effects analysis of treatment group, time and interaction on primary outcomes.

Outcome	Most severe ideation^a^					Suicide behaviors^a^					Suicidal thoughts^b^				
	Beta (SE)	*p*	CI (lower to higher)	*χ* ^2^ (df)	*p*	Beta (SE)	*p*	CI (lower to higher)	*χ* ^2^ (df)	*p*	OR (SE)	*p*	CI (lower to higher)	*χ* ^2^ (df)	*p*
Full model															
Treatment	0.496 (1.021)	0.627	(−1.505 to 2.498)	0.94 (3)	0.817	0.263 (0.835)	0.752	(−1.372 to 1.899)	3.14 (3)	0.371	0.649 (1.072)	0.686	(−2.532 to 1.666)	18.29 (4)	0.011
Time	−0.001 (0.008)	0.959	(−0.016 to 0.015)			−0.033 (0.027)	0.227	(−0.087 to 0.021)			1.007 (0.011)	0.521	(−0.015 to 0.029)		
Treatment × Time	−0.006 (0.011)	0.549	(−0.027 to 0.015)			0.022 (0.029)	0.443	(−0.034 to 0.078)			0.966 (0.016)	0.030*	(−0.066 to 0.003)		

Outcome	Depression^c^					Medication utilization^d^					School services utilization^d^				
	Beta (SE)	*p*	CI (lower to higher)	*χ* ^2^ (df)	*p*	OR (SE)	*p*	CI (lower to higher)	*χ* ^2^ (df)	*p*	OR (SE)	*p*	CI (lower to higher)	*χ* ^2^ (df)	*p*
Full model															
Treatment	−0.858 (1.065)	0.421	(−2.945 to 1.230)	42.30 (6)	< 0.001	1.545 (0.875)	0.619	(−1.279 to 2.150)	24.23 (4)	< 0.001	0.630 (0.518)	0.372	(−1.478 to 0.553)	32.57 (4)	< 0.001
Time	−0.015 (0.008)	0.067	(−0.030 to 0.001)			0.969 (0.010)	0.001**	(−0.051 to −0.013)			0.961 (0.010)	< 0.001**	(−0.031 to 0.020)		
Treatment × Time	−0.007 (0.010)	0.483	(−0.026 to 0.012)			1.004 (0.011)	0.686	(−0.017 to 0.025)			1.010 (0.011)	0.397	(−0.013 to 0.032)		

Variable	Inpatient services^a^					Outpatient services^a^				
	OR (SE)	*p*	CI (lower to higher)	*χ* ^2^ (df)	*p*	OR (SE)	*p*	CI (lower to higher)	*χ* ^2^ (df)	*p*
Full model										
Treatment	1.649 (0.575)	0.394	(−0.638 to 1.617)	14.32 (3)	0.003	1.351 (0.793)	0.704	(−1.252 to 1.855)	2.86 (3)	0.413
Time	0.963 (0.019)	0.041*	(−0.075 to −0.002)			0.992 (0.008)	0.327	(−0.024 to 0.008)		
Treatment × Time	0.989 (0.023)	0.657	(−0.055 to 0.035)			0.999 (0.010)	0.983	(−0.020 to 0.019)		

^a^ No covariates were significantly related to outcome; thus, none were included in the model.

^b^ Race was controlled for as a significant covariate in the model.

^c^ Sex, Race and sexual gender minority were controlled for as significant covariates in the model.

^d^ Age was controlled for as a significant covariate in the model.

* Significant at the 0.05 level.

** Significant at the 0.01 level.

**FIGURE 2:**
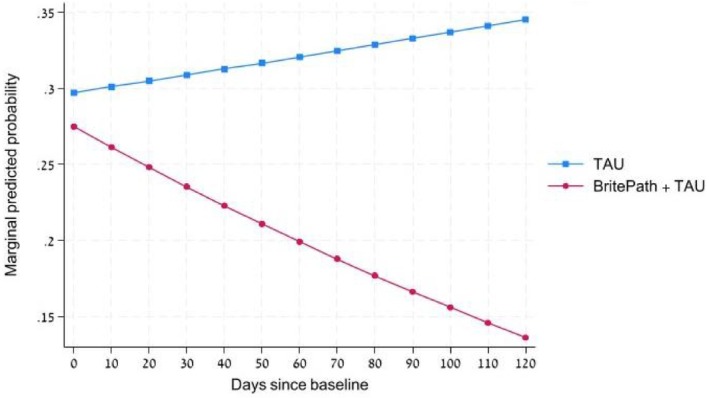
Treatment group by time interaction on suicidal thoughts. Marginal predicted probability of suicidal thoughts.

A significant treatment group by time interaction (OR = 0.966, SE = 0.016, *p* = 0.030) was obtained on suicidal thoughts (item 9 on the PHQ‐9), while controlling for significantly related covariates. This indicated that over time, there was a lower probability of endorsing suicidal thoughts in BRITEpath + TAU compared to TAU (see Figure 2). No significant main effects for time (OR = 1.007, SE = 0.011, *p* = 0.0.521) and treatment group (OR = 0.649, SE = 1.072, *p* = 0.686) were observed. In terms of QOL, there were no significant differences between treatment groups in all sub‐scores and total score of the QOL at weeks 4 and 12 (see Table S5).

**FIGURE 3:**
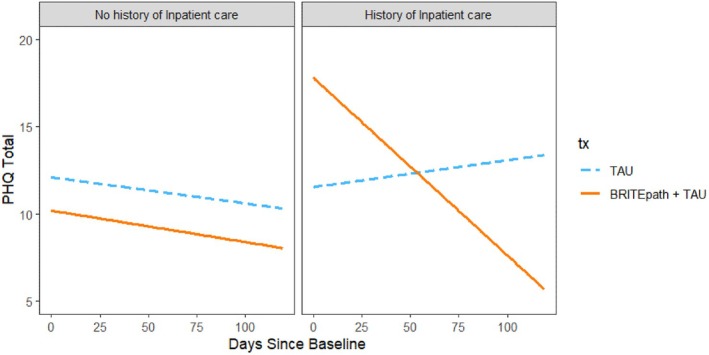
Moderation effect of history of inpatient care on depression over time.

**FIGURE 4:**
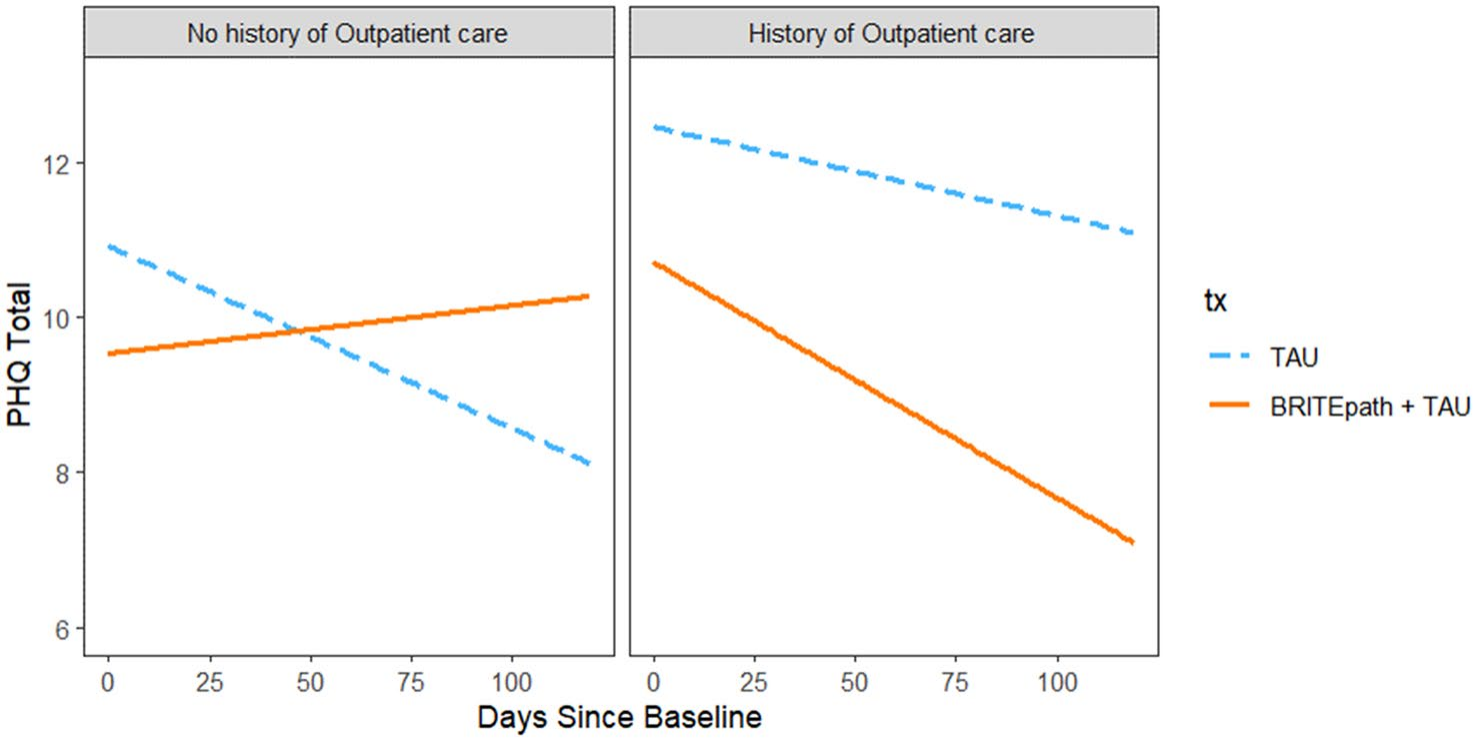
Moderation effect of history of outpatient care on depression over time.

Moderation analysis revealed that a history of inpatient care moderated the effect of treatment on depression over time, with a stronger effect of BRITEpath + TAU among those who had a history of inpatient care (*β* = −0.114, SE = 0.052, *p* = 0.029) (see Figure 3). A similar moderation pattern was observed for a history of outpatient care, revealing a stronger effect of BRITEpath + TAU on the decrease of depression over time (*β* = −0.049, SE = 0.023, *p* = 0.031) among those with a history of outpatient care (see Figure 4).

### 3.3 Secondary Outcomes

#### 3.3.1 Application Utilization

Table S6 shows the number of participants who used each activity of the BRITE app. The most commonly used activity was the distress rating; 37 participants (54.1%) completed their 1st distress rating, and 42.6% (*n* = 29) completed the second distress ratings, which were used an average of 14.65 (SD = 13.73, range 1–50, median = 10) times per participant. Crisis survival strategies were the second most commonly used activity (20.6%, *n* = 14), with an average use of 3.14 (SD = 3.10, range 1–11, median = 2) times per participant. This was followed by distraction techniques (19.1%, *n* = 13) with an average use of 1.62 (SD = 1.33, range 1–5, median = 1) times per participant. Guided meditation was the least used feature (13.2%, *n* = 9), with an average use of 2.22 (SD = 1.79, range 1–6, median = 1) times per participant. Overall, 37 (54.1%) and 29 (42.6%) participants used at least one and more than one activity in the app, respectively. There were no significant differences between those who used the app to those who did not on primary outcomes (see Table S7).

#### 3.3.2 Application Usability and Satisfaction

The average usability score on each item ranged from 4.56 to 5.32, with higher scores indicating higher usability ratings. On satisfaction items, 68.7% of participants reported that they would recommend BRITEPath for people in need of mental health services and 58.8% rated 3 for satisfaction with the amount of help received from BRITEpath (on a scale from 1‐very dissatisfied to 4‐very satisfied) (see Table 3). Most participants (74.0%) reported that BRITEpath helped in dealing with their emotional problems.

**TABLE 3:** Application usability and satisfaction—mean ratings of satisfaction and frequency of responses on satisfaction items.

Usability	Overall *N*	Mean (SD)
Satisfied w/how easy to use	50	4.72 (2.00)
Information for coping was clear	40	5.15 (2.06)
Information to rate distress was clear	40	5.02 (2.12)
Information during onboarding was clear	40	5.32 (2.03)
Liked interacting with Brite	50	4.56 (1.91)

Satisfaction	Overall *N*	*N* (%) of participants rating each response
Needed to learn before get going w/Brite	51	
Strongly Disagree		27 (52.9%)
2		14 (27.5%)
3		6 (11.8%)
4		2 (3.9%)
Strongly Agree		2 (3.9%)
Would recommend Brite	51	
No, definitely not		8 (15.7%)
No, I don't think so		8 (15.7%)
Yes, I think so		19 (37.3%)
Yes, definitely		16 (31.4%)
Satisfaction w/amount of help received	51	
Very Dissatisfied		5 (9.8%)
2		6 (11.8%)
3		30 (58.8%)
Very Satisfied		10 (19.6%)
Services helped dealing w/problems	51	
Seemed to make things worse		2 (4.0%)
Really didn't help		11 (22.0%)
Yes, somewhat		26 (52.0%)
Yes, a great del		11 (22.0%)

#### 3.3.3 Rapid Content Analysis

Responses to open‐ended questions were obtained from *N* = 51 participants. Table 4 represents the frequency of responses that included any reference for each domain and theme, including both positive and negative comments. Twenty‐two (43.1%) participants commented on the perceived usability of different features and resources offered by the application, with most indicating that they found them useful (*N* = 21, 41.2%). However, others indicated that they perceived the application was less appropriate to their needs (*N* = 20, 39.2%), either because it provided them with skills they were already familiar with, or they thought that the application would be more appropriate as a supplement for conventional mental health treatment (like psychotherapy).

**TABLE 4:** Frequency^a^ of responses relating to each domain and theme from rapid content analysis.

Domains/Themes	*N*	(%)	Exemplar quotes
Perceived Usability (Domain 1)			
Ease of use	11^b^	21.6	“[The] app was easy to use”
Ease of access	10^c^	19.6	“[It was] helpful to have something easy to get to.”
Problems with use	18	35.3	“I found that the app had some glitches in terms of daily dose and I found the suggested activities were difficult to navigate and not that helpful” “for one, i didn't get any notifications to update the daily feedback or daily dose. [The app] didn't tell me to interact with it so I just forgot.”
Usefulness of app features	22^d^	43.1	“Likes how it was organized; Provides information that you would need if you are distressed; Provides resources. Likes that the app provides useful phone numbers. Likes the mood tracker and other resources that can help”
Perceived Feasibility (Domain 2)			
Adherence issues	10	19.6	“More on my part to remember to use it, I didn't remember to use it” “I just wish I put in more work to remember to use it. I just wish it had better notifications to remind me to use it” “Didn't use it enough—didn't feel like i was very intrigued by it”
Perceived Acceptability (Domain 3)			
Satisfaction with application	16^e^	31.4	“Got more than they expected from the study/app.”
Perceived appropriateness	23^f^	45.1	“Gave a lot of good skills but many of skills and resources were things I already tried.” “I would recommend a therapist, someone who can monitor more closely and have human interactions, brite app would be a good tool to add in addition”
Perceived Efficacy (Domain 4)			
Help in general	22^g^	43.1	“Learning coping skill was helpful”
Help in moments of stress	5	9.8	“The app helped me when I was stressed and helped me cope.”
Help with symptoms	4	7.8	“Used the app during ‘episodes of depression and anxiety’, the app helped to manage and organize resources to support during those times.”

^a^ Frequencies represent any responses (whether positive or negative) that relate to a specific theme.

^b^ *N* = 9 had positive comments, and *N* = 2 had negative comments on ease of use.

^c^ *N* = 9 had positive comments, and *N* = 1 had negative comments on ease of access.

^d^ *N* = 21 had positive comments, and *N* = 1 had negative comments on usefulness of app features.

^e^ *N* = 13 had positive comments, and *N* = 3 had negative comments on satisfaction with app.

^f^ *N* = 3 had positive comments, and *N* = 20 had negative comments on perceived appropriateness.

^g^ *N* = 17 had positive comments, and *N* = 5 had negative comments on whether the app provided help in general.

## 4 Discussion

This RCT examined the feasibility and preliminary efficacy of a primary care‐based smartphone intervention, BRITEpath, on suicide‐ and depression‐related outcomes. Findings indicate no significant differences between the treatment groups in most primary outcomes. However, those assigned to BRITEpath had a lower frequency of suicidal thoughts over time compared to TAU, reflecting an effect on self‐injurious ideation. In addition, moderation analysis revealed that BRITEpath had a stronger effect on depression over time among those who had a history of inpatient or outpatient care.

While our findings are inconsistent with two previous studies of BRITE, which demonstrated positive, albeit not always statistically significant, effects on reducing the hazard of subsequent suicide attempts following discharge among adolescents in inpatient care (Goldstein et al. 2024; Kennard et al. 2018), they nonetheless offer important insights. The following section outlines key differences between the current and prior studies that may account for the discrepancy in findings and highlights lessons learned and recommendations for future research.

Participants in previous BRITE studies were recruited following admission to psychiatric inpatient units for a recent suicide attempt or SI with plan and/or intent (Goldstein et al. 2024; Kennard et al. 2018), placing them at significantly higher suicide risk than the current sample, which was recruited in primary care. Youth screened in primary care are generally at lower suicide risk compared to those discharged from inpatient units or EDs (Davis et al. 2024; Braciszewski et al. 2023), and may be less forthcoming about SI (Flores et al. 2024). Our participants had a low incidence of SBs (4.4%) and many were already receiving mental health services prior to and during the study (69.3%). In contrast, the rate of suicide attempts in TAU in our previous two studies was 31% and 17.5%, respectively (Goldstein et al. 2024; Kennard et al. 2018). More importantly, the groups did not differ in their utilization of mental health services prior and during the study. As such, participants were already receiving ongoing support, which may have attenuated the ability to detect incremental effects of BRITEpath beyond the level of care that they were already receiving. This may also have reduced the perceived relevance or added value of BRITEpath. In addition, participants in the TAU group received repeated assessments of suicidality by the research team during follow‐up visits, with referral and additional safety planning provided as needed, which may have enhanced monitoring and support beyond usual care and consequently attenuated suicide risk in this group, thereby reducing detectable differences between treatment arms.

Notably, BRITEpath appeared more effective in reducing depression among youth with a history of inpatient care in the current study, consistent with earlier BRITE findings showing greater impact among adolescents with prior suicide attempts (Goldstein et al. 2024; Kennard et al. 2018). These results suggest BRITE, as currently configured, may be most effective for higher‐risk youth, highlighting the need to tailor BRITE for varying risk levels and care settings to enhance its broader applicability (Kruzan et al. 2024).

In contrast to participants in previous BRITE studies (Goldstein et al. 2024; Kennard et al. 2018), who received at least two follow‐up coaching calls to support BRITE use, participants in our sample were contacted only for follow‐up assessments after their initial primary care visit (Tighe et al. 2017; Stallard et al. 2024). Coaching calls included motivational interviewing strategies to address challenges and barriers to engagement with the app, as well as inquiries about the skills obtained from the app (Kennard et al. 2018). This makes a considerable difference in application engagement and uptake implementation of app use, especially among adolescents vulnerable to suicide (Bailey et al. 2024). Thus, future iterations of BRITEpath can incorporate human support (e.g., coaching calls and follow‐ups with clinicians) to enhance patient engagement.

Previous studies in digital mental health highly recommended the use of digital applications in conjunction with human contact either alongside therapeutic treatment or through coaching calls (Nwosu et al. 2022). This is supported by our participants' recommendations that the app will be integrated with in‐person contact and/or as a supplement to psychotherapy and provide reminder notifications to use the app. Additionally, in the current study, BRITEpath was not utilized by MH clinicians in their ongoing care, underscoring the need to adapt future digital tools to clinical workflows and provider needs. While provider input was included within the design of BRITEpath, future iterations should further incorporate providers' considerations toward workflow to assure it can feasibly be implemented within routine care and aligns with their preferences, a critical step that could increase uptake and sustained engagement (Lyon et al. 2019). In addition, BRITEpath could be further tailored to address the needs of patients across varying levels of suicide risk and adapted for implementation across diverse clinical settings that provide crisis intervention at different levels of care, including emergency departments, inpatient units, outpatient services, and community‐ or school‐based settings.

Despite the limited efficacy of BRITEpath on treatment outcomes, post‐study surveys indicated moderate satisfaction, and most found the app easy to use. Distress ratings were the most used feature, suggesting relevance for adolescents vulnerable to suicide. Those results correspond with prior findings from BRITE (Goldstein et al. 2024; Kennard et al. 2018), reflecting similar patterns of use. In addition, studies examining other suicide‐related digital health interventions highlighted the importance of distress and mood monitoring in raising self‐awareness and mitigating emotional distress among adolescents vulnerable to suicide (Sarubbi et al. 2022). Participants also reported that BRITE facilitated their coping mechanisms when facing emotional distress, and that they would recommend it to a friend who might be in need of mental health care. This corresponds to findings from other suicide‐related digital health tools, where satisfaction from apps is not always reflected in changes in clinical outcomes (Sutori et al. 2024). This may suggest that changes in clinical outcomes from stand‐alone digital health interventions are limited, perhaps requiring some contact with MH clinicians.

These findings should be interpreted in light of several limitations. First, generalizability is limited, as the sample was predominantly female (85.1%), White (70.3%), and only 7% Hispanic. Although the primary care setting was chosen to enhance diversity, future studies should adopt more active strategies to recruit ethnically diverse youth, such as employing a diverse clinical team and offering culturally tailored digital interventions. Second, due to the pilot nature of the study, limited data on certain outcomes (e.g., quality of life, ER admissions) precluded more robust longitudinal analyses. Additionally, due to the pilot nature of the study, adjustment for multiple outcomes was not conducted. Third, the sample spanned a wide age range (12–26), which may be a limitation, as adolescents and young adults likely have differing needs from digital health tools. Incorporating developmentally tailored features may help address this in future research.

## 5 Conclusion

Taken together, our findings suggest that BRITEpath may be beneficial in reducing suicidal thoughts and is more effective in alleviating depression among participants with a history of inpatient or outpatient care, with participants reporting favorable usability and satisfaction. However, its limited impact on other outcomes may be due to minimal integration with human contact and its application among lower‐risk adolescents in primary care settings who were already receiving mental health services. Adolescents and young adults might require more frequent human contact to facilitate and encourage app use, especially in primary care settings. We assert recommendations made previously (Nwosu et al. 2022; Garrido et al. 2019), that engagement and efficacy of digital health platforms is facilitated by integrating apps with ongoing care.

## Author Contributions

**Giovanna Porta:** data curation, formal analysis, writing – review and editing, methodology. **Kaleab Z. Abebe:** conceptualization, formal analysis, writing – review and editing. **Casey Monteverde:** formal analysis, data curation. **Jamie Zelazny:** writing – review and editing, methodology, conceptualization, investigation. **Stephanie Stepp:** supervision, writing – review and editing, methodology, conceptualization. **David Brent:** writing – review and editing, conceptualization, investigation, funding acquisition, methodology, supervision. **Brandie George‐Milford:** conceptualization, investigation, data curation, writing – review and editing, project administration. **Nermin Toukhy:** writing – original draft, formal analysis. **Candice Biernesser:** conceptualization, methodology, writing – review and editing.

## Funding

This work was supported by the National Institute of Mental Health (MH115838), the Israeli Science Foundation (grant no. 50/24), the Fulbright fellowship program, and the University of Pittsburgh.

## Ethics Statement

All the study procedures were approved by the institutional review board of the University of Pittsburgh under protocol #'s: STUDY20110359, STUDY20050381, and STUDY19050132.

## Consent

Participants and their legal guardians signed an informed consent form; an informed assent was provided by participants under the age of 18. The informed consent forms included permission to use survey data.

## Conflicts of Interest

Dr. Brent has reported receiving grants from NIMH (supported the development of intellectual property for the BRITE app, the As Safe As Possible intervention), the American Foundation for Suicide Prevention (AFSP), Once Upon a Time Foundation, and The Beckwith Institute; receiving royalties from Guilford Press, eRT Inc., for the electronic self‐rated version of the Columbia Suicide Severity Rating Scale, and UptoDate; receiving honoraria from the Klingenstein Third Generation Foundation for scientific board membership and grant review. The other authors declare no conflicts of interest.

## Supporting information


**Table S1:** Consort checklist: CONSORT 2010 checklist of information.
**Table S2:** Codebook for rapid content analysis.
**Table S3:** Sociodemographic and clinical characteristics by participants with vs. without follow‐up data.
**Table S4:** Group comparisons between BRITEPath and TAU in mental health services utilization at baseline and follow‐ups.
**Table S5:** Group comparisons between Britepath and TAU in QOL sub‐scores and total scores at Week 4 and Week 12.
**Table S6:** Application utilization—number of participants completing application features.
**Table S7:** Group comparisons between those who did vs. didn't use the BRITE app.

## Data Availability

The data that support the findings of this study are available through the NIMH Data Archive (NDA) website at https://nda.nih.gov.
