# Supplementary material for: Examining BRITEpath, a Digital Intervention for Reducing Adolescent Suicide Risk in Primary Care: A Randomized‐Controlled Trial
Source: Suicide Life Threat Behav. 2026 Jun 9;56(3):e70114. doi: 10.1111/sltb.70114 (PMC13247626; doi:10.1111/sltb.70114)
Supplement: Supplementary file 1 — Table S1: Consort checklist: CONSORT 2010 checklist of information. Table S2: Codebook for rapid content analysis. Table S3: Sociodemographic and clinical characteristics by participants with vs. without follow‐up data. Table S4: Group comparisons between BRITEPath and TAU in mental health services utilization at baseline and follow‐ups. Table S5: Group comparisons between Britepath and TAU in QOL sub‐scores and total scores at Week 4 and Week 12. Table S6: Application utilization—number of participants completing application features. Table S7: Group comparisons between those who did vs. didn't use the BRITE app. [file SLTB-56-0-s001.docx]

**Supplementary Table 1: Consort Checklist:** **CONSORT 2010 checklist of information**

| **Section/Topic** | **Item No** | **Checklist item** | **Reported on:** |
| --- | --- | --- | --- |
| **Title and abstract** | | | |
|  | 1a | Identification as a randomised trial in the title | Title |
|  | 1b | Structured summary of trial design, methods, results, and conclusions (for specific guidance see CONSORT for abstracts) | Abstract |
| **Introduction** | | | |
| Background and objectives | 2a | Scientific background and explanation of rationale | Introduction, paragraphs 1 - 5 |
|  | 2b | Specific objectives or hypotheses | Introduction, paragraph 6 |
| **Methods** | | | |
| Trial design | 3a | Description of trial design (such as parallel, factorial) including allocation ratio | Methods, paragraph 5 |
|  | 3b | Important changes to methods after trial commencement (such as eligibility criteria), with reasons | Methods, paragraph 8, 9 |
| Participants | 4a | Eligibility criteria for participants | Methods, paragraph 1, 2 |
|  | 4b | Settings and locations where the data were collected | Methods, paragraphs 1, 2 |
| Interventions | 5 | The interventions for each group with sufficient details to allow replication, including how and when they were actually administered | Methods, paragraphs 6-9, 19, 20 |
| Outcomes | 6a | Completely defined pre-specified primary and secondary outcome measures, including how and when they were assessed | Methods, paragraphs 10-18 |
|  | 6b | Any changes to trial outcomes after the trial commenced, with reasons | N/A |
| Sample size | 7a | How sample size was determined | Methods, paragraph 22 |
|  | 7b | When applicable, explanation of any interim analyses and stopping guidelines | N/A |
| Randomisation: |  |  |  |
| Sequence generation | 8a | Method used to generate the random allocation sequence | Methods, paragraph 5 |
|  | 8b | Type of randomisation; details of any restriction (such as blocking and block size) | Methods, paragraph 5 |
| Allocation concealment mechanism | 9 | Mechanism used to implement the random allocation sequence (such as sequentially numbered containers), describing any steps taken to conceal the sequence until interventions were assigned | Methods, paragraph 5 |
| Implementation | 10 | Who generated the random allocation sequence, who enrolled participants, and who assigned participants to interventions | Methods, paragraphs 19, 20 |
| Blinding | 11a | If done, who was blinded after assignment to interventions (for example, participants, care providers, those assessing outcomes) and how | Methods, paragraph 20 |
|  | 11b | If relevant, description of the similarity of interventions | Methods, paragraph 9 |
| Statistical methods | 12a | Statistical methods used to compare groups for primary and secondary outcomes | Methods, paragraph 21-24 |
|  | 12b | Methods for additional analyses, such as subgroup analyses and adjusted analyses | Methods, paragraphs 21-24 |
| **Results** | | | |
| Participant flow (a diagram is strongly recommended) | 13a | For each group, the numbers of participants who were randomly assigned, received intended treatment, and were analysed for the primary outcome | Results, paragraph 1 |
|  | 13b | For each group, losses and exclusions after randomisation, together with reasons | Results, paragraph 1 |
| Recruitment | 14a | Dates defining the periods of recruitment and follow-up | Results, paragraph 1 |
|  | 14b | Why the trial ended or was stopped | N/A |
| Baseline data | 15 | A table showing baseline demographic and clinical characteristics for each group | Results, Table 1 |
| Numbers analysed | 16 | For each group, number of participants (denominator) included in each analysis and whether the analysis was by original assigned groups | Results, Figure 1 |
| Outcomes and estimation | 17a | For each primary and secondary outcome, results for each group, and the estimated effect size and its precision (such as 95% confidence interval) | Results, paragraphs 3-5; Table 2 |
|  | 17b | For binary outcomes, presentation of both absolute and relative effect sizes is recommended | Results, paragraphs 3-5; Table 2 |
| Ancillary analyses | 18 | Results of any other analyses performed, including subgroup analyses and adjusted analyses, distinguishing pre-specified from exploratory | Results, paragraphs 5-8 |
| Harms | 19 | All important harms or unintended effects in each group (for specific guidance see CONSORT for harms) | N/A |
| **Discussion** | | | |
| Limitations | 20 | Trial limitations, addressing sources of potential bias, imprecision, and, if relevant, multiplicity of analyses | Discussion, Paragraph 6 |
| Generalisability | 21 | Generalisability (external validity, applicability) of the trial findings | Discussion, Paragraph 6 |
| Interpretation | 22 | Interpretation consistent with results, balancing benefits and harms, and considering other relevant evidence | Discussion, Paragraph 6 |
| **Other information** | | |  |
| Registration | 23 | Registration number and name of trial registry | Methods, paragraph 1 |
| Protocol | 24 | Where the full trial protocol can be accessed, if available | Methods, paragraph 1 |
| Funding | 25 | Sources of funding and other support (such as supply of drugs), role of funders | Funding Statement |

*We strongly recommend reading this statement in conjunction with the CONSORT 2010 Explanation and Elaboration for important clarifications on all the items. If relevant, we also recommend reading CONSORT extensions for cluster randomised trials, non-inferiority and equivalence trials, non-pharmacological treatments, herbal interventions, and pragmatic trials. Additional extensions are forthcoming: for those and for up to date references relevant to this checklist, see [www.consort-statement.org](http://www.consort-statement.org/).

| **Supplementary Table 2: Codebook for Rapid Content Analysis** | | |
| --- | --- | --- |
| **Main domain definition** | **Theme** | **Definition** |
| **Usability** |  |  |
| Defined as the participants’ perceptions of using the BRITE app, including their emotions, values, and motivations (Inal et al., 2020). | *Ease of use* | Participants' perception of how easy the BRITE app was to use |
|  | *Ease of access* | Participants' perception of how accessible was the contents of the BRITE app |
|  | *Problems with use* | Participants' perception of any issues and challenges they faced while using the app, including technical-related (app getting stuck, glitches, problems with onboarding) or user-related (difficulty in using certain features of the app) issues |
|  | *Usefulness of app features* | Participants' perception of how useful they found the different contents/features of the app |
| **Feasibility** |  |  |
| Defined as pariticipants' perceptions of whether the BRITE app can be used in their ongoing care | *Adherence issues* | Reports regarding whether participants did not use the app during the study, including forgetting to use the app. If participants specify a certain barrier to use related to specific features of the app- code as "Problems with use" |
| **Acceptability** |  |  |
| Defined as pariticipants' perceptions of satisfcation with BRITE app and whether the BRITE app was appropriate to their needs | *Satisfaction with app* | Participants' perception of how satisfied they are with the app |
|  | *Perceived appropriateness* | Participants' perception of whether (or not) the features/content offered by BRITE app were appropriate for them (i.e. how much participants perceived that the interventions offered by the app was relevant and suitable for them). |
| **Efficacy** |  |  |
| Defined as whether participants perceived that the app was efficient in helping them with their needs | *Help in general* | Participants' perceptions of whether the app helped them in general (i.e. without stating how and with what the app helped them) |
|  | *Help in moments of stress and crises* | Participants' perceptions of whether the app helped them during moments of emotional distress and crises |
|  | *Help with symptoms (depression, anxiety, SIBs)* | Participants' perceptions of whether the app helped them with any symptoms that they have- e.g. anxiety, depression, panic attacks and suicide thoughts and behaviors |
| **Other** |  |  |
| Participants’ perspectives that did not fall into the sub-domains mentioned above |  | Paricipants' suggestions for app improvements and whether the app would be helpful for others |

**Supplementary Table 3: Sociodemographic and clinical characteristics by participants with vs. without follow-up data**

|  | With follow-up data | Without follow-up data | Statistic | p-value |
| --- | --- | --- | --- | --- |
| N | 89 | 12 |  |  |
| **Sociodemographic characteristics** |  |  |  |  |
| Group condition= BRITEpath | 58 (65.2%) | 10 (83.3%) | χ²(1) = 1.59 | 0.208 |
| Age (cont) (range 12-26) | 19.10 (3.78) | 20.00 (3.46) | t(99) = -0.78 | 0.437 |
| Age (>18) | 50 (56.2%) | 9 (75.0%) | χ²(1) = 0.86 | 0.353 |
| Sex (Male) | 12 (13.5%) | 3 (25.0%) | χ²(1) = 0.39 | 0.535 |
| Race (White) | 62 (69.7%) | 9 (75.0%) | χ²(1) = 0.14 | 0.704 |
| Family income (>75,000$) | 22 (43.1%) | 3 (37.5%) | χ²(1) = 0 | 1.000 |
| SGM | 50 (56.2%) | 5 (41.7%) | χ²(1) = 0.41 | 0.523 |
| **Clinical Characteristics** |  |  |  |  |
| Depression (phq total) | 10.52 (5.95) | 13.33 (4.50) | t(99) = -1.58 | 0.118 |
| Physical QOL (pedsq-physical) | 77.63 (20.55) | 82.29 (11.83) | t(29) = -0.38 | 0.705 |
| Emotional QOL (pedsql-emotional) | 56.79 (25.47) | 50.00 (18.03) | t(29) = 0.45 | 0.659 |
| Social QOL (pedsql-social) | 75.98 (25.84) | 65.00 (13.23) | t(29) = 0.72 | 0.478 |
| School QOL (pedsql-school) | 60.36 (24.94) | 51.67 (10.41) | t(29) = 0.59 | 0.559 |
| Psychosocial QOL (pedsql) | 64.30 (21.40) | 55.56 (11.10) | t(29) = 0.69 | 0.495 |
| Overall QOL | 68.93 (19.86) | 64.86 (11.10) | t(29) = 0.35 | 0.731 |
| **Suicide Thoughts and Behavior ^a^** |  |  |  |  |
| Suicidal thoughts (phq item 9) | 0.45 (0.78) | 0.67 (0.89) | t(99) = -0.89 | 0.377 |
| Most severe ideation | 1.17 (1.56) | 1.67 (1.97) | t(99) = -1.01 | 0.317 |
| Preparatory acts (CSSRS) | 1 (1.1%) | 1 (9.1%) | χ²(1) = 0.41 | 0.523 |
| Aborted attempt (CSSRS) | 2 (2.2%) | 1 (9.1%) | χ²(1) = 0.1 | 0.750 |
| Interrupted attempt (CSSRS) | 2 (2.2%) | 0 (0.0%) | χ²(1) = 0 | 1.000 |
| Actual attempt (CSSRS) | 5 (5.6%) | 0 (0.0%) | χ²(1) = 0.01 | 0.942 |
| Any Suicidal Behavior | 7 (7.9%) | 1 (9.1%) | χ²(1) = 0 | 1.000 |
| **Service Utilization ^b^** |  |  |  |  |
| Outpatient Services | 80 (89.9%) | 11 (100.0%) | χ²(1) = 0.3 | 0.584 |
| School Services | 52 (58.4%) | 6 (54.5%) | χ²(1) = 0 | 1.000 |
| Emergency Services | 14 (15.7%) | 2 (18.2%) | χ²(1) = 0 | 1.000 |
| Legal Services | 1 (1.1%) | 0 (0.0%) | χ²(1) = 0 | 1.000 |
| Inpatient Services | 22 (24.7%) | 3 (27.3%) | χ²(1) = 0 | 1.000 |
| Medication Services | 75 (85.2%) | 10 (90.9%) | χ²(1) = 0 | 0.959 |
| ^a^ Past-3 months Suicidal Thoughts and Behaviors assessed at baseline  ^b^ Past-3 months Service utilization assessed at baseline | | | | |

**Supplementary Table 4: Group comparisons between BRITEPath and TAU in mental health services utilization at baseline and follow-ups**

|  | BRITEPath | TAU | Statistic | p-value |
| --- | --- | --- | --- | --- |
| Mental health services at baseline^a^ | 65 (97.0%) | 31 (93.9%) | χ²(1) = 0.55 | 0.461 |
| Mental health services at week 4 | 52 (83.9%) | 29 (90.6%) | χ²(1) = 0.81 | 0.369 |
| Mental health services at week 12 | 51 (86.4%) | 26 (81.3%) | χ²(1) = 0.43 | 0.512 |
| ^a^ Past-3 months medication, inpatient, outpatient or school mental health service utilization assessed at baseline | | | | |

**Supplementary Table 5: Group comparisons between Britepath and TAU in QOL sub-scores and total scores at week 4 and week 12**

|  | ***N*** | **Overall** | **BRITEPath** | **TAU** | **Test** | **Statistic** | ***p*** | **Effect size (Hedge’s g)** |
| --- | --- | --- | --- | --- | --- | --- | --- | --- |
| **Quality of Life** |  |  |  |  |  |  |  |  |
| Physical QOL week 4 | 28 | 83.59(17.59) | 89.31(11.52) | 71.53(22.45) | t(26) | -2.240 | 0.024 | -1.098 |
| Physical QOL week 12 | 28 | 82.92(17.81) | 87.17(15.20) | 73.96(20.43) | t(26) | -1.923 | 0.033 | -0.755 |
| Emotional QOL week 4 | 28 | 66.07(24.28) | 68.68(25.87) | 60.56(20.83) | t(26) | -0.822 | 0.209 | -0.323 |
| Emotional QOL week 12 | 28 | 59.46(24.43) | 63.68(25.43) | 50.56(20.68) | t(26) | -1.348 | 0.095 | -0.530 |
| Social QOL week 4 | 28 | 79.82(24.32) | 81.58(24.10) | 76.11(25.83) | t(26) | -0.548 | 0.294 | -0.215 |
| Social QOL week 12 | 28 | 79.29(28.08) | 82.63(27.61) | 72.22(29.38) | t(26) | -0.913 | 0.185 | -0.359 |
| School QOL week 4 | 28 | 66.07(22.25) | 70.26(22.51) | 57.22(20.02) | t(26) | -1.480 | 0.075 | -0.581 |
| School QOL week 12 | 28 | 68.93(23.58) | 72.11(23.47) | 62.22(23.73) | t(26) | -1.037 | 0.155 | -0.407 |
| Psychosocial QOL week 4 | 28 | 70.65(21.10) | 73.51(21.57) | 64.63(19.89) | t(26) | -1.041 | 0.154 | -0.409 |
| Psychosocial QOL week 12 | 28 | 69.23(21.62) | 72.81(23.22) | 61.67(16.44) | t(26) | -1.289 | 0.104 | -0.506 |
| Overall QOL week 4 | 28 | 75.16(18.32) | 79.00(16.40) | 67.03(20.44) | t(26) | -1.667 | 0.054 | -0.655 |
| Overall QOL week 12 | 28 | 73.99(18.98) | 77.8(19.32) | 65.94(16.40) | t(26) | -1.587 | 0.062 | -0.623 |

**Supplementary Table 6: Application Utilization – number of participants completing application features**

| **App feature- completed** | **Overall N** | **Num completed (%)** |
| --- | --- | --- |
| 1^st^ distress level | 68 | 37(54.4%) |
| 2^nd^ distress level | 68 | 29(42.6%) |
| Crisis survival strategies | 68 | 14(20.6%) |
| Distraction exercise | 68 | 13(19.1%) |
| Distract happy thoughts | 68 | 17(25.0%) |
| Learn to savor | 68 | 4(5.9%) |
| Reaching out to contacts | 68 | 11(16.2%) |
| Used savor | 68 | 15(22.1%) |
| Used soothe activities | 68 | 10(14.7%) |
| Used soothe breathe | 68 | 17(25.0%) |
| Used soothe guided meditation | 68 | 9(13.2%) |
| Used +1 activity* | 68 | 29(42.6%) |

**Supplementary Table 7: Group comparisons between those who did vs. didn’t use the BRITE app**

|  | **Did not use BRITE app** | **Did use BRITE app** | **Test** | **p** | **Statistic** | **Effect size (Cohen’s d/ Cramer’s V)** |
| --- | --- | --- | --- | --- | --- | --- |
| **N** | **31** | **37** |  |  |  |  |
| **Primary outcomes at follow-up** | | | | | |  |
| Depression at week 4 (mean (SD)) | 8.40 (4.57) | 8.81 (6.32) | t | 0.781 | -0.279 | -0.072 |
| Depression at week 12 (mean (SD)) | 8.78 (5.02) | 7.94 (5.64) | t | 0.564 | 0.580 | 0.153 |
| Physical QOL at week 4 (mean (SD)) | 92.19 (7.44) | 88.54 (12.48) | t | 0.589 | 0.551 | 0.296 |
| Physical QOL at week 12 (mean (SD)) | 82.81 (20.17) | 88.33 (14.25) | t | 0.534 | -0.635 | -0.341 |
| Emotional QOL at week 4 (mean (SD)) | 68.75 (17.50) | 68.67 (28.19) | t | 0.996 | 0.006 | 0.003 |
| Emotional QOL at week 12 (mean (SD)) | 71.25 (13.15) | 61.67 (27.82) | t | 0.519 | 0.659 | 0.354 |
| Social QOL at week 4 (mean (SD)) | 82.50 (23.63) | 81.33 (25.03) | t | 0.934 | 0.084 | 0.047 |
| Social QOL at week 12 (mean (SD)) | 88.75 (22.50) | 81.00 (29.29) | t | 0.632 | 0.488 | 0.262 |
| School QOL at week 4(mean (SD)) | 83.75 (16.52) | 66.67 (22.96) | t | 0.185 | 1.382 | 0.743 |
| School QOL at week 12 (mean (SD)) | 85.00 (17.80) | 68.67 (24.09) | t | 0.226 | 1.256 | 0.675 |
| Psychosocial QOL at week 4 (mean (SD)) | 78.33 (11.55) | 72.22 (23.69) | t | 0.629 | 0.493 | 0.116 |
| Psychosocial QOL at week 12 (mean (SD)) | 81.67 (15.69) | 70.44 (24.74) | t | 0.406 | 0.852 | 0.458 |
| Overall QOL at week 4 (mean (SD)) | 83.15 (9.07) | 77.90 (17.95) | t | 0.584 | 0.558 | 0.300 |
| Overall QOL at week 12 (mean (SD)) | 82.07 (17.15) | 76.67 (20.25) | t | 0.633 | 0.486 | 0.261 |
| **Suicide ideation and behaviors** | | | | | |  |
| Self-harm thoughts (phq item 9) at week 4 | 0.36 (0.76) | 0.24 (0.64) |  | 0.258 | 0.654 | 0.690 |
| Self-harm thoughts (phq item 9) at week 12 | 0.26 (0.54) | 0.17 (0.45) |  | 0.235 | 0.727 | 0.485 |
| Most severe suicide ideation at week 4(mean (SD)) | 0.72 (1.14) | 0.97 (1.54) | t | 0.485 | -0.703 | -0.180 |
| Most severe suicide ideation at week 12 (mean (SD)) | 0.87 (1.46) | 0.42 (1.00) | t | 0.199 | 1.309 | 0.374 |
| Suicide Behaviors at week 4 (%) | 0 (0) | 1 (2.7%) | 𝜒2(1) | 0.407 | 0.687 | 0.105 |
| Suicide Behaviors at week 12 (%) | 2 (8.7%) | 1 (2.8%) | 𝜒2(1) | 0.313 | 1.018 | -0.131 |
| **Utilization of services** | | | | | |  |
| Medication treatment at week 4 (%) | 16 (64.0) | 21 (56.8) | 𝜒2(1) | 0.568 | 0.325 | -0.072 |
| Medication treatment at week 12 (%) | 16 (69.6) | 18 (50.0) | 𝜒2(1) | 0.138 | 2.200 | -0.193 |
| Legal services at week 4 (%) | 0 (0) | 0 (0) | 𝜒2(1) | NA | NA | NA |
| Legal services at week 12(%) | 0 (0) | 0 (0) | 𝜒2(1) | NA | NA | NA |
| ER admissions at week 4(%) | 0 (0.0) | 1 (2.7) | 𝜒2(1) | 0.407 | 0.687 | 0.105 |
| ER admissions at week 12 (%) | 0 (0) | 0 (0) | 𝜒2(1) | NA | NA | NA |
| School services at week 4 (%) | 1 (4.0) | 7 (18.9) | 𝜒2(1) | 0.086 | 2.955 | 0.218 |
| School services at week 12 (%) | 3 (13.0) | 5 (13.9) | 𝜒2(1) | 0.926 | 0.009 | 0.012 |
| Inpatient services at week 4 (%) | 0 (0.0) | 2 (5.4) | 𝜒2(1) | 0.237 | 1.396 | 0.150 |
| Inpatient services at week 12 (%) | 0 (0.0) | 1 (2.8) | 𝜒2(1) | 0.420 | 0.650 | 0.105 |
| Outpatient services at week 4 (%) | 18 (72.0) | 28 (75.7) | 𝜒2(1) | 0.746 | 0.105 | 0.041 |
| Outpatient services at week 12 (%) | 17 (73.9) | 32 (88.9) | 𝜒2(1) | 0.135 | 2.236 | 0.195 |
